# An Artificial Intelligence Approach to the Assessment of Abnormal Lid Position

**DOI:** 10.1097/GOX.0000000000003089

**Published:** 2020-10-27

**Authors:** Peter B. M. Thomas, Chrishan D. Gunasekera, Swan Kang, Tadas Baltrusaitis

**Affiliations:** From the * NIHR Biomedical Research Centre for Ophthalmology, Moorfields Eye Hospital NHS Foundation Trust and UCL Institute of Ophthalmology, London, United Kingdom; † Norfolk and Norwich University Hospital NHS Foundation Trust, Colney Ln, Norwich, United Kingdom; ‡ Moorfields Eye Hospital NHS Foundation Trust, London, United Kingdom; § Microsoft, London, United Kingdom.

## Abstract

New artificial intelligence (AI) approaches to facial analysis show promise in the clinical evaluation of abnormal lid position. This could allow more naturalistic, quantitative, and automated assessment of lid position. The aim of this article was to determine whether OpenFace, an AI approach to real-time facial landmarking and analysis, can extract clinically useful measurements from images of patients before and after ptosis correction. Manual and AI-automated approaches to vertical palpebral aperture measurement of 128 eyes in pre- and postoperative full-face images of ptosis patients were compared in this study. Agreement in interpupillary distance to vertical palpebral aperture ratio between clinicians and an AI-based system was assessed. Image quality varied highly with interpupillary distance defined by a mean of 143.4 pixels (min = 60, max = 328, SD = 80.3 pixels). A Bland–Altman analysis suggests a good agreement between manual and AI analysis of vertical palpebral aperture (94.4% of measurements falling within 2 SDs of the mean). Correlation between the 2 methods yielded a Pearson’s r(126) = 0.87 (*P* < 0.01) and r^2^ = 0.76. This feasibility study suggests that existing, open-source approaches to facial analysis can be applied to the clinical assessment of patients with abnormal lid position. The approach could be extended to further quantify clinical assessment of oculoplastic conditions.

## INTRODUCTION

Automation of medical care has been seriously suggested for over 50 years.^1^ The early attempts at automation through artificial intelligence (AI) failed to translate to clinical practice—they automated clinical tasks that could be rapidly performed by the clinician and failed to automate other tasks that remained clinician-dependent. Perceptual judgments, for example interpretation of radiology images, have remained solidly the domain of human clinicians until recently.

Over the past few years, developments in AI, most notably deep learning, have significantly increased the capability of machines to simulate human-like perceptual judgments. In medicine, this has most dramatically been demonstrated in the grading of diabetic retinopathy^2^ and the diagnosis of skin cancer.^3^ In these examples, the images used for diagnosis are static and highly homogeneous. Some perceptual judgments, however, require the clinician to interpret a visual object that is mobile, under non-ideal lighting, and at an uncontrolled distance, for example interpretation of facial abnormalities in a non-compliant child. Other judgments are intrinsically dynamic—for example, interpretation of a blink reflex or of facial muscle function.

Traditionally, computer vision has not coped well with faces—they are complex, moving, and highly variable visual objects. In ophthalmology we are often confronted with patients complaining of ptosis (drooping eyelids). Determining the best management of ptosis relies on measurement of parameters, including the marginal reflex distances (distance between a corneal light reflex and upper lid margin, MRD1, and lower lid margin, MRD2), palpebral aperture (maximum vertical distance between upper and lower lid margin), and levator function (distance the upper eyelid travels from downgaze to upgaze). The traditional approach is to measure these using a ruler. Manual analysis can be performed on patient photographs using computer software.^4,5^ Bespoke automated image analysis tools have been developed for the express purpose of assessing eyelid positions.^6–8^ However, these methods require specialized software environments, and high quality, controlled patient photographs. Assessment of the lid height in a dynamic face with different visual tasks would help determining how much the lid should be lifted surgically.

In this article, we present a feasibility study of the use of an open source AI-driven facial analysis system, OpenFace,^9,10^ for clinical facial analysis. We have previously shown a related system to be equivalent to gold-standard for the measurement of abnormal head posture, a task that predominantly relies on detection of landmarks defined by bones.^11^ We now analyze its performance at recognizing a feature defined entirely by soft tissue: the palpebral aperture.

## METHODS

In this feasibility study, we use a task-modified version of OpenFace to measure palpebral aperture in publicly available pre- and postoperative photographs of ptosis surgery, and compare it with the assessment of 2 human graders. Principles outlined in the Declaration of Helsinki have been followed.

### Facial Images

Images of 32 patients with ptosis were gathered using a Google search. These comprised a preoperative and postoperative image for each patient, and both eyes were included in the analysis. No limitation was placed on the resolution of the image, though only images showing the full face of the patient were used.

### Manual Assessment of Palpebral Aperture

Palpebral aperture was assessed independently by 2 assessors using standard image software (Paint.net and ImageJ). The interpupillary distance (IPD) and vertical palpebral aperture were measured using the calliper tool and expressed in pixels. The assessors were instructed to measure the vertical maximum height of the palpebral aperture. The ratio of the palpebral aperture measured in this way to the IPD was calculated and used as the comparator metric for palpebral aperture.

### Automated Measurement of Palpebral Aperture

Automated measurement of palpebral aperture was performed in OpenFace. The image is first analyzed using Histogram of Oriented Gradient and Support Vector Machine techniques to detect faces. A simplified model of the face is then used to constrain the regions in which to search for 68 facial landmarks. Landmarks were identified using a machine learning technique called “constrained local neural fields”. Pixel coordinates of lid margins and corneal limbi are then used to calculate the ratio of vertical palpebral aperture to interpupillary distance.

## RESULTS

Image quality varied widely with interpupillary distance defined by as few as few as 60 pixels and as many as 328 (mean = 143.4, SD = 80.3 pixels).

**Fig. 1.**
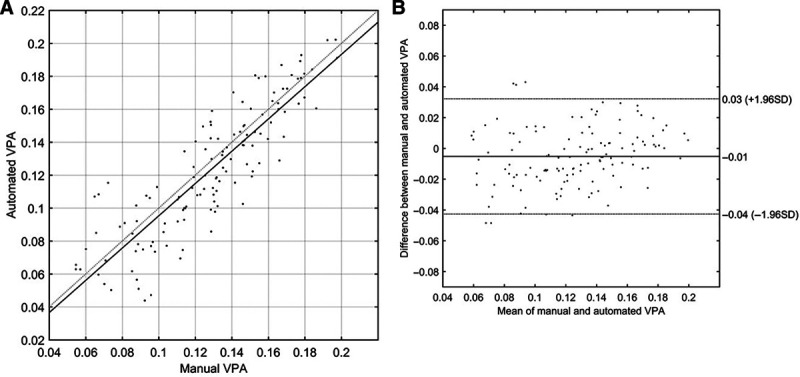
A, A comparison of vertical palpebral aperture (VPA), defined as (vertical palpebral aperture height in pixels)/(interpupillary distance in pixels), between the automated (AI) and manual method. The solid line is the line of best fit, and the dotted line defines a line of gradient 1 and passing through the origin. B, A Bland–Altman analysis of the automated (AI) and manual methods. The abscissa shows the average VPA for each eye across the 2 methods, while the ordinate shows the difference in VPA between the 2 methods. The lines show the positions of the mean difference and ±1.96 SD.

Comparison between the automated and manual (averaged across the 2 assessors) metric of vertical palpebral aperture gives a Pearson’s r(126) = 0.87 (*P* < 0.01), r^2^ = 0.76 (see Fig. 1A). Figure 1B shows a Bland–Altmann analysis of the automated and manual methods. An estimated 94.4% of all observations fall within 2 SDs of the mean, suggesting a good agreement.

## DISCUSSION

In this preliminary work, open source AI-based facial analysis software was able to perform to a good level of agreement with manual human assessment of palpebral aperture. Performance was relatively robust across a range of palpebral apertures and image resolutions, and without careful control of head position or image lighting.

The work presented here has clear limitations. First, we measure only the palpebral aperture. For detailed ptosis assessment, other values would also have to be measured, including the marginal reflex distance, levator function, and brow position. We were also unable to measure the absolute value of palpebral apertures but used the ratio of IPD to vertical palpebral aperture as a proxy. Further work will be undertaken on high-quality clinical images to improve the performance of the system and expand its capabilities to detect other clinically useful features, including measurements of marginal reflex distances. Improved resolution and image quality can be expected to improve performance compared with the current study.

The technology has potential to enhance the clinical assessment of ptosis. The main strength of this algorithm is its ability to measure the lid height using photographs and videos without specialized hardware or software environments. This technology could be incorporated in telemedicine modules, where digital photographs or videos are taken by nonprofessionals, including the patients themselves. The dynamic assessment of the eyelids in videos could enable the clinicians to appreciate the change in lid height while carrying out different visual tasks.
